# Vulnerable Waters are Essential to Watershed Resilience

**DOI:** 10.1007/s10021-021-00737-2

**Published:** 2022-02-07

**Authors:** Charles R. Lane, Irena F. Creed, Heather E. Golden, Scott G. Leibowitz, David M. Mushet, Mark C. Rains, Qiusheng Wu, Ellen D’Amico, Laurie C. Alexander, Genevieve A. Ali, Nandita B. Basu, Micah G. Bennett, Jay R. Christensen, Matthew J. Cohen, Tim P. Covino, Ben DeVries, Ryan A. Hill, Kelsey Jencso, Megan W. Lang, Daniel L. McLaughlin, Donald O. Rosenberry, Jennifer Rover, Melanie K. Vanderhoof

**Affiliations:** 1 Office of Research and Development, U.S. Environmental Protection Agency, 980 College Station Road, Athens, Georgia 30605, USA;; 2 Department of Physical and Environmental Sciences, University of Toronto Scarborough, 1065 Military Trail, Toronto, Ontario M1C 1A4, Canada;; 3 Office of Research and Development, U.S. Environmental Protection Agency, 26 W. Martin Luther King Dr., Cincinnati, Ohio 45268, USA;; 4 Office of Research and Development, Center for Public Health and Environmental Assessment, Pacific Ecological Systems Division, U.S. Environmental Protection Agency, 200 SW 35th Street, Corvallis, Oregon 97333, USA;; 5 Northern Prairie Wildlife Research Center, U.S. Geological Survey, 8711 37th Street Southeast, Jamestown, North Dakota 58401, USA;; 6 School of Geosciences, University of South Florida, 4202 E. Fowler Avenue, NES 107, Tampa, Florida 33620, USA;; 7 Department of Geography, University of Tennessee, 309 Burchfiel Geography Building, Knoxville, Tennessee 37996, USA;; 8 Office of Research and Development, Pegasus Technical Services, c/o U.S. Environmental Protection Agency, 26 W. Martin Luther King Dr., Cincinnati, Ohio 45268, USA;; 9 Office of Research and Development, U.S. Environmental Protection Agency, 1200 Pennsylvania Avenue, Washington, District of Columbia 20460, USA;; 10 School of Environmental Sciences, University of Guelph, 50 Stone Road East, Guelph, Ontario N1G 2W1, Canada;; 11 Civil and Environmental Engineering and Earth and Environmental Studies, University of Waterloo, 200 University Avenue West, Waterloo, Ontario N2L 3G1, Canada;; 12 U.S. Environmental Protection Agency, Region 5, 77 W. Jackson Boulevard, Chicago, Illinois 60604, USA;; 13 School of Forest, Fisheries, and Geomatics Sciences, University of Florida, 118 Newins-Ziegler Hall, PO Box 110410, Gainesville, Florida 32611, USA;; 14 Department of Ecosystem Science and Sustainability, Warner College of Natural Sciences, Colorado State University, 1476 Campus Delivery, Fort Collins, Colorado 80523, USA;; 15 Department of Geography, Environment, and Geomatics, University of Guelph, 50 Stone Road East, Guelph, Ontario N1G 2W1, Canada;; 16 Montana Climate Office, W.A. Franke College of Forestry and Conservation, University of Montana, 32 Campus Drive, Missoula, Montana 59812, USA;; 17 U.S. Fish and Wildlife Service, National Wetlands Inventory, 5275 Leesburg Pike, Falls Church, Virginia 22041, USA;; 18 Department of Forest Resources and Environmental Conservation, Virginia Tech, 201C Cheatham Hall, 310 West Campus Drive, Blacksburg, Virginia 24061, USA;; 19 U.S. Geological Survey, Water Mission Area, W 6th Avenue, Kipling Street, Lakewood, Colorado 80225, USA;; 20 Earth Resources Observation and Science Center, U.S. Geological Survey, 47914 252nd Street, Sioux Falls, South Dakota 57198, USA;; 21 Geosciences and Environmental Change Science Center, U.S. Geological Survey, MS 980, PO Box 25046, Denver, Colorado 80225, USA

**Keywords:** ephemeral stream, geographically isolated wetlands, headwater stream, intermittent river and ephemeral stream, intermittent stream, non-floodplain wetland, perennial stream, state transitions, steady state, thresholds, water quality, watershed management

## Abstract

Watershed resilience is the ability of a watershed to maintain its characteristic system state while concurrently resisting, adapting to, and reorganizing after hydrological (for example, drought, flooding) or biogeochemical (for example, excessive nutrient) disturbances. Vulnerable waters include non-floodplain wetlands and headwater streams, abundant watershed components representing the most distal extent of the freshwater aquatic network. Vulnerable waters are hydrologically dynamic and biogeochemically reactive aquatic systems, storing, processing, and releasing water and entrained (that is, dissolved and particulate) materials along expanding and contracting aquatic networks. The hydrological and biogeochemical functions emerging from these processes affect the magnitude, frequency, timing, duration, storage, and rate of change of material and energy fluxes among watershed components and to downstream waters, thereby maintaining watershed states and imparting watershed resilience. We present here a conceptual framework for understanding how vulnerable waters confer watershed resilience. We demonstrate how individual and cumulative vulnerable-water modifications (for example, reduced extent, altered connectivity) affect watershed-scale hydrological and biogeochemical disturbance response and recovery, which decreases watershed resilience and can trigger transitions across thresholds to alternative watershed states (for example, states conducive to increased flood frequency or nutrient concentrations). We subsequently describe how resilient watersheds require spatial heterogeneity and temporal variability in hydrological and biogeochemical interactions between terrestrial systems and down-gradient waters, which necessitates attention to the conservation and restoration of vulnerable waters and their downstream connectivity gradients. To conclude, we provide actionable principles for resilient watersheds and articulate research needs to further watershed resilience science and vulnerable-water management.

## Introduction

Vulnerable waters, that is, headwater streams (ephemerally, intermittently, and perennially flowing, low-order, lotic waters) and non-floodplain wetlands (also called geographically isolated wetlands, Leibowitz 2003), perform critical watershed functions that affect the magnitude, frequency, timing, duration, and rate of change of material and energy fluxes among watershed components and to downstream waters (Ward 1989; Cadenasso and others 2003; Loreau and others 2003; Rains and others 2016; Brooks and others 2018; Gómez-Gener and others 2021). These functions include extensive biogeochemical processing (Arce and others 2019; Golden and others 2019) and substantive hydrological flood attenuation and baseflow maintenance (Hubbard and Linder 1986; Evenson and others 2016; Fossey and Rousseau 2016; Golden and others 2021).

The term vulnerable waters emerged due to their susceptibility to degradation or destruction because of the insufficiency of their mapped extent and limited regulatory protection (see Creed and others 2017). Yet vulnerable waters are often abundant watershed components within natural landscapes (Freeman and others 2007; Lane and D’Amico 2016; Allen and others 2018; Hafen and others 2020; Fesenmyer and others 2021; Messager and others 2021), with estimates suggesting they comprise up to 89% of longitudinal stream extent worldwide (Allen and others 2018) and greater than 16% of inland wetlands in the conterminuous USA (Lane and D’Amico 2016); no global estimates exist for non-floodplain wetlands.

Though their individual and cumulative contributions are increasingly noted in the scientific literature (for example, McGuire and others 2014; Marton and others 2015; Cohen and others 2016; Rains and others 2016; Creed and others 2017; Cheng and others 2020; Kim and Park 2020), the potentially controlling effects of vulnerable waters on watershed-scale ecological resilience (hereafter watershed resilience) has not yet been fully considered by the scientific community. Watershed resilience is the ability of a watershed to maintain conditions, functions, structures, interactions, and feedbacks (that is, maintain its characteristic system state) while concurrently resisting, recovering from, adapting to, and reorganizing after disturbances (Holling 1973; Walker and others 2004; Ives and Carpenter 2007). Disturbances affecting watershed resilience come in both acute, short-term, reorganizational shocks (for example, fires, floods) and chronic, long-term stresses (for example, increasing urbanization, changing precipitation regimes) (Moloney and Levin 1996; Stanford and others 2005; Kleindl and others 2015).

Watershed resilience is informed by the state and interactions of a watershed’s terrestrial and aquatic components in both the built and natural environments, including wetlands, streams, rivers, lakes, forests, grasslands, urban areas, and agricultural lands (Hynes 1975; Forman 1995). Resilient watersheds withstand and adapt to disturbances prior to functional or structural thresholds being crossed (Scheffer and others 2001). However, once thresholds are crossed, watersheds undergo a regime shift resulting in measurable and marked change in state-defining storages, process rates, and interactions (Folke and others 2004; Carpenter and others 2011). Watershed resilience brings an important geospatially bounded and increasingly resource management focused perspective to the broader ecological resilience concept (Murphy and Sprague 2019).

A watershed’s state and resilience can be determined by hydrological and biogeochemical storages and fluxes to the aquatic system that emanate from vulnerable waters through their interactions with their terrestrial drainage areas and contributing near-surface and groundwater flow networks (Larned and others 2010; Sayer 2014; Covino 2017; Hare and others 2021). Through their cumulative effects on down-gradient material and energy fluxes (Biggs and others 2017), vulnerable waters control (for example, dampen) hydrological and biogeochemical variability at watershed outlets (Saco and Kumar 2002; Lindsay and others 2004; Cohen and others 2016; Rupp and others 2021).

However, humans are decreasing watershed resilience and contributing to watershed state changes through disturbances that modify vulnerable-water extent and hydrological and biogeochemical functioning (for example, Dahl 1990; Elmore and Kaushal 2008; Wright and Wimberly 2013; Van Meter and Basu 2015; Johnston and McIntyre 2019). For instance, alterations to vulnerable waters have been implicated as possible causal agents in down-gradient nutrient-mediated lake trophic-state regime shifts (for example, Engstrom and others 2006; Zhang and others 2009). Golden and others (2021) reported that watershed-scale hydrological modification of vulnerable-water storage functions affected down-gradient stream discharge, increasing the magnitude, frequency, duration, and related impacts of flooding. Coupling hydrological and biogeochemical disturbances, watershed-scale drainage modifications (for example, tiling) affected storage and fluxes from vulnerable waters, altered stream hydrographs and increased materials (for example, nutrients) entrained in modified drainages across the upper Midwestern United States, leading to watershed-scale ecohydrological state shifts (Foufoula-Georgiou and others 2015; McKenna and others 2017).

Modifications of vulnerable waters alter the time-integrated and spatially disparate relationships between precipitation and conversion to flowing water in streams (for example, McKenna and others 2017). The mechanisms of these alterations that modify resilience and catalyze state changes are straightforward: hydrologically concentrating (through headwater stream channelization), dissipating (through non-floodplain wetland drainage), and bypassing (for example, through agricultural tiles, Gramlich and others 2018; or urban piping, Elmore and Kaushal 2008) vulnerable waters decrease their storage capacity and changes their contributions to the variability of down-gradient streamflow. Concurrently, alterations to vulnerable waters that expedite hydrological travel times decrease biogeochemically important residence times while increasing nutrient loading to down-gradient aquatic systems (Golden and others 2019), negating vulnerable-water functioning as biogeochemical hot spots for nutrient assimilation (for example, Marton and others 2015; Cheng and Basu 2017; Cheng and others 2020; Evenson and others 2021).

Sufficiently widespread and impactful disturbances to vulnerable waters may lead to a regime shift, transforming the watershed across thresholds of measured function or structure into an alternative state (Zelnik and Meron 2018). Once a transition to a new state occurs, a new suite of watershed descriptors will emerge with stable structures and defined functions, processes, and interactions (Botkin and Sobel 1975; Angeler and Allen 2016; McKenna and others 2017; Mushet and others 2020). The new post-disturbance state, like the old, has definable watershed resilience and will similarly withstand disturbances. However, the post-transition state may be societally undesirable (Scheffer and others 2001; Allen and others 2016). Further, changing watershed states through restoration requires energetically demanding, resource consuming, or otherwise policy-constricting modifications to overcome the resilience of the new state (Biggs and others 2009). Hence, maintenance of crucial system structures, functions, and the resulting time-varying interactions between terrestrial land covers and vulnerable waters is important for watershed resilience (Ward 1989; Saco and Kumar 2002; Uden and others 2014; Cohen and others 2016).

Here, we present a novel framework linking the functions of vulnerable water to the maintenance of watershed state and the resilience of watersheds, building on recent reviews (for example, Larned and others 2010; Cohen and others 2016; Rains and others 2016; Wohl 2017; Fritz and others 2018; Crabot and others 2021). Our intended audience includes researchers functionally linking headwater streams and non-floodplain wetlands with watershed-scale hydrological and biogeochemical phenomena (for example, Cheng and others 2020; Evenson and others 2021), as well as natural resource managers who are increasingly adopting watershed-scale perspectives and practices to address vexing societal water quality and quantity problems (Creed and others 2017; Accatino and others 2018). We first describe the concept of watershed resilience. Second, we review the scientific literature describing vulnerable-water effects on watershed state-defining hydrological and biogeochemical storages and fluxes affecting resilience. Third, we characterize how modifying vulnerable-water extent and functions decreases watershed resilience, which can precipitate a state change. We follow in the fourth section by articulating vulnerable-water emergent theories and management principles for judiciously guarding and improving watershed state and increasing watershed resilience. We conclude by identifying research and management needs for improved watershed resilience science.

## Watershed Resilience

**Figure 1.**
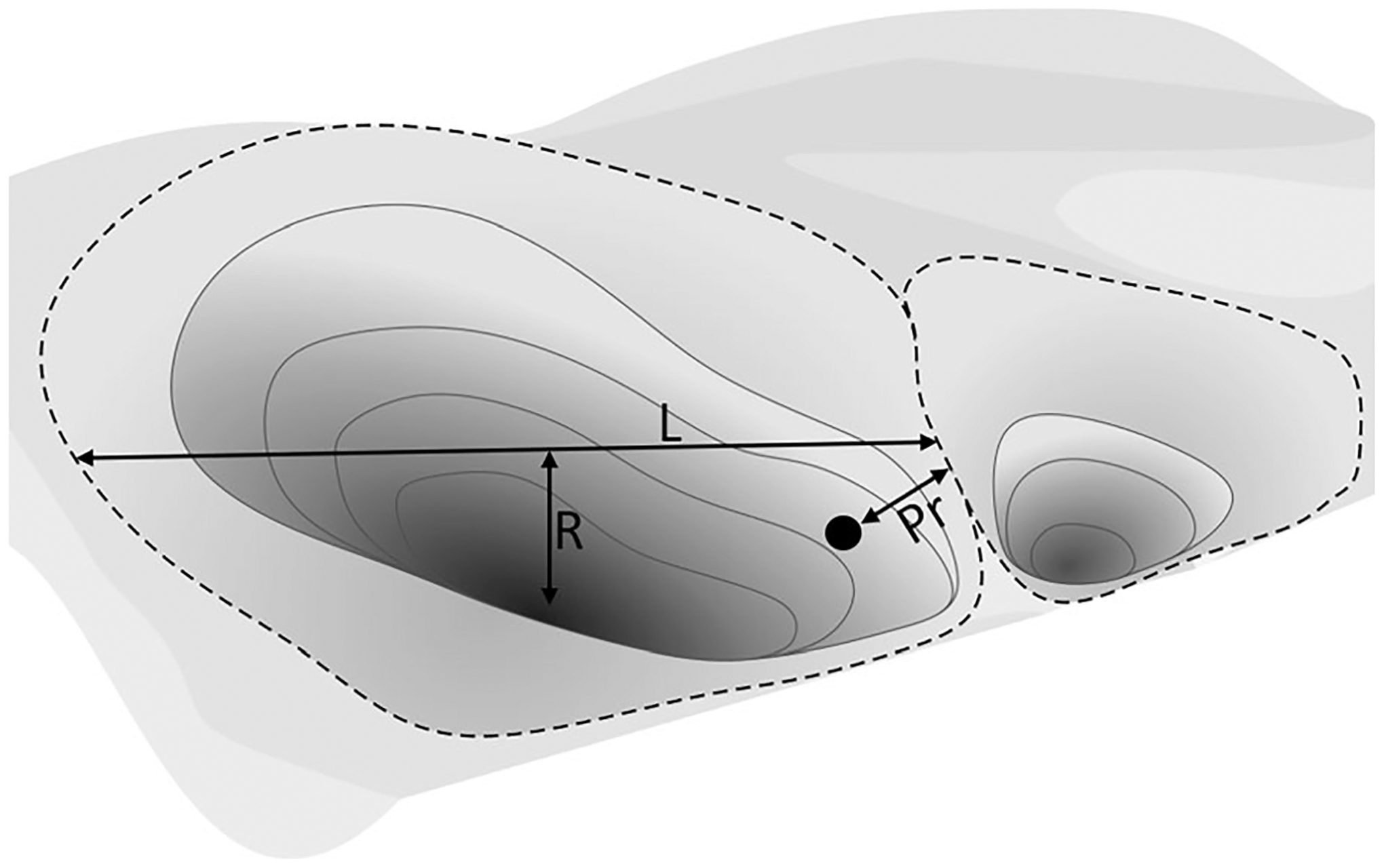
Watershed states viewed as a conceptual landscape of current and possible depression-like states or “basins of attraction” demarcated by dashed boundary lines. The resilience of each watershed state is defined by the resistance to change (*R*, or the depth of the basin), latitude to disturbance (*L*, or the width of the basin), and precariousness (Pr, or the proximity of the current state to the state change threshold). A given watershed’s current state is indicated by the black dot, which we posit represents the measurable hydrological (for example, flood attenuation and baseflow maintenance) and biogeochemical (for example, biogeochemical processing and flux-magnitude dampening) functions performed and affected by an existing suite of vulnerable waters and their down-gradient connectivity, quantified at the watershed outlet. Transitions to alternative watershed states, represented by proximal basins of attraction, are hypothesized to occur with destruction of extant vulnerable waters and/or diminution of their functions. Modified from Walker and others (2004) and used under Creative Commons Attribution/Non-Commercial 4.0 International License.

Watershed resilience is a concept founded on ecological resilience and the existence of multiple alternative watershed states (Holling 1973; Rinaldi and Scheffer 2000). Each alternative watershed state is defined by structural and functional characteristics and their resultant hydrological and biogeochemical processes (for example, storages and fluxes) as measured at the watershed outlet. Like a watershed’s current state, each alternative state has resilience to change (Beisner and others 2003). Watershed resilience is described by several theoretical descriptors (Walker and others 2004; Figure 1). *Latitude* describes the width of the state-space, or the amount a watershed can be altered before transiting across a threshold to a new watershed state. *Resistance* is the depth of the basin of attraction, or relative effort needed to change the watershed to a new state*. Precariousness* characterizes the proximity of the watershed state to a threshold after which a transition to a different basin of attraction will occur (Walker and others 2004; Zipper and others 2020). Resilient watersheds are conceptually located in basins of attraction with wide latitude and deep resistance, both of which confer the ability to withstand greater disturbances prior to undergoing a regime shift or crossing a threshold to a different watershed state (see Figure 1; Menck and others 2013; Radchuk and others 2019). Similarly, resilient watersheds are those distant from precarious thresholds, thereby less likely to cross a change threshold (that is, undergo a transformative regime shift to a new stable state with defined structures and functions affecting hydrological and biogeochemical storages and fluxes in response to a given disturbance; Sasaki and others 2015).

Vulnerable waters convey watershed resilience by dampening disturbance effects on hydrological and biogeochemical storages and fluxes while concurrently enforcing feedbacks that strengthen the provisioning of characteristic watershed functions (that is, vulnerable waters provide “balancing” or “self-regulating” feedbacks). In resilience parlance, hydrologically and biogeochemically mediated functions performed by vulnerable waters provide *negative* feedbacks that convey watershed resilience for a given watershed state (that is, adaptability and resistance to change). Conversely, when vulnerable waters are impacted, these hydrologically and biogeochemically mediated functions are altered, leading to the loss of these negative feedbacks or even the creation of positive feedbacks (that is, “reinforcing” the coming watershed change) that accelerate transitions across thresholds to alternative watershed states.

**Figure 2.**
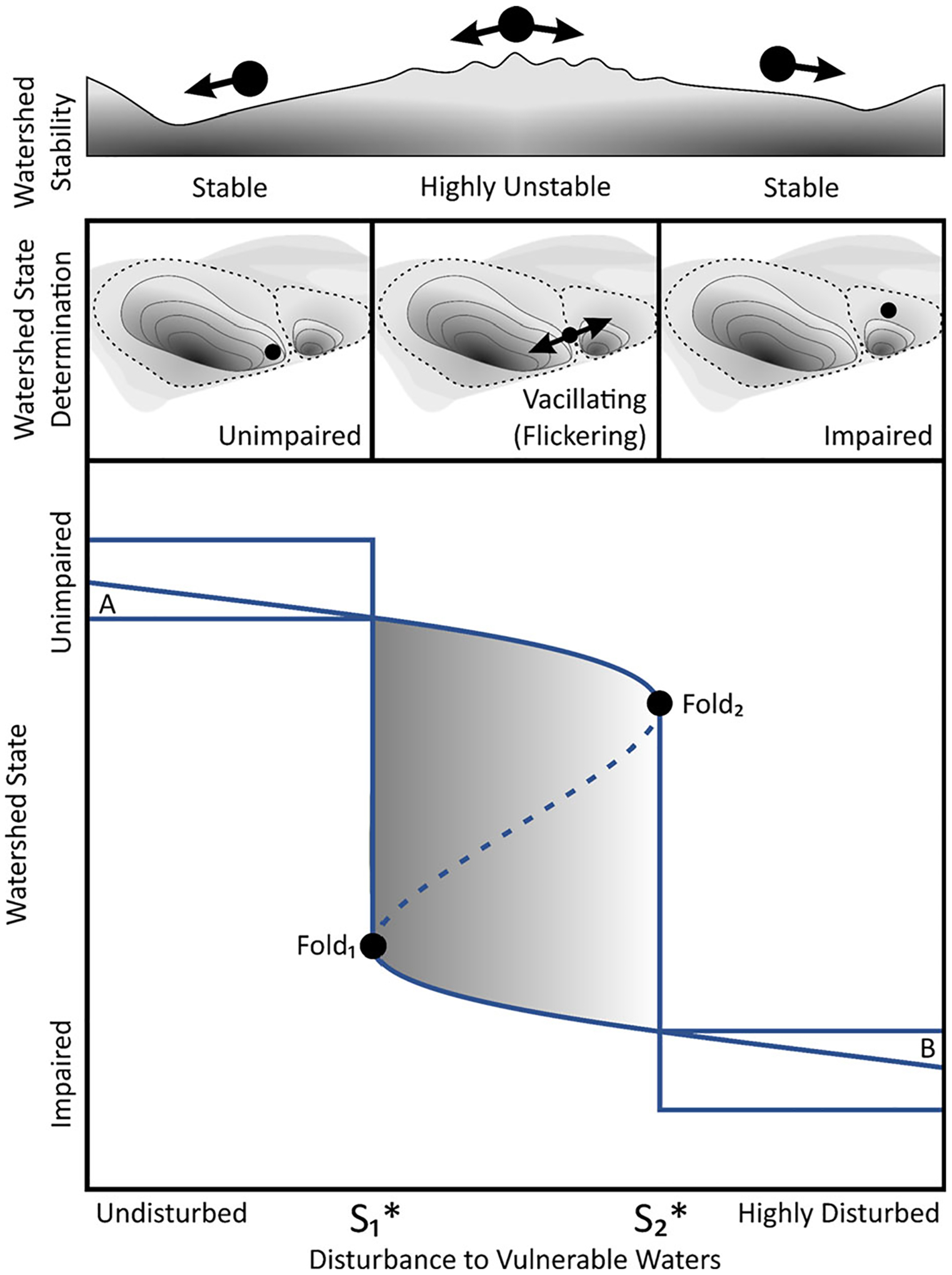
Properly functioning and network-connected vulnerable waters determine watershed state. Incremental and/or cumulative disturbances to vulnerable waters < *S*_1_* (Fold_1_ in the bottom graph) affect watershed state yet not to the degree to precipitate a state-changing transition (that is, hydrological and biogeochemical functioning and network disturbance dampening provided by vulnerable waters maintains watershed state). Following Rinaldi and Scheffer (2000), at disturbance values < *S*_1_*, the watershed state (for example, as measured by nutrient loads) remains in the unimpaired basin of attraction as indicated by black dot within the dashed basin boundary in the middle diagram (and within Box A in the bottom diagram). Hence, vulnerable-water disturbances < *S*_1_* are insufficient to disrupt the inherent state stability and steady-state dynamics of watershed’s basin of attraction (top diagram). However, vulnerable-water disturbance levels in the bottom diagram ≥ *S*_1_* and ≤ *S*_2_* result in highly unstable states (top diagram) wherein the watershed may vacillate or flicker over time between either state (middle box of middle diagram). These unstable equilibria may presage state transitions (Scheffer and others 2012). Continued disturbances to vulnerable waters transit the watershed across a threshold (Fold_2_, disturbance values > *S*_2_*) to a new and stable steady state. For example, consider a watershed experiencing disturbance in the form of nutrient loading. On the *y*-axis of the bottom diagram, the watershed system as hypothetically qualified by given nutrient loads at a pour point is stable, and in an unimpaired state with vulnerable-water disturbance < *S*_1_*, bi-stable or flickering between ≥ *S*_1_* and ≤ *S*_2_*, and stable in an impaired state when disturbance values > *S*_2_.

As regime shifts loom, unstable watershed states may emerge (Rinaldi and Scheffer 2000). Like a swaying tightrope walker, watersheds may enter a liminal and unstable state with different and vacillating (or “flickering”) states emergent at any point in time (Figure 2). However, once a characteristic state-defining threshold is crossed, the watershed enters a new basin of attraction as it undergoes a transformational change in structures, functions, and feedbacks (for example, decreased hydrological yields affecting stream flow, temperature regimes, and habitat; Hicks and others 1991). Subsequent to a threshold being crossed, a watershed then emerges in a stable watershed state, with new structural, functional, and interactive characteristics—including different system resilience and disturbance thresholds (Scheffer and others 2012)—that define the watershed’s hydrological and biogeochemical state.

## Vulnerable Waters Contribute TO Watershed Resilience

Increased scientific interest in vulnerable-water functions has emerged concurrent with both new policy and management challenges (for example, Lassaletta and others 2010; Alexander 2015; Colvin and others 2019; Mihelcic and Rains 2020) and the burgeoning availability of high spatial and temporal resolution data (for example, Vanderhoof and Lane 2019; Wu and others 2019a). Several recent reviews detail the state of the science on the individual and cumulative functional effects of headwater streams and non-floodplain wetlands on downstream system states (for example, Larned and others 2010; Marton and others 2015; USEPA 2015; Cohen and others 2016; Rains and others 2016; Biggs and others 2017; Golden and others 2017, 2019; Wohl 2017; Fritz and others 2018; Lane and others 2018; Schofield and others 2018; Gómez-Gener and others 2021). Those in-depth reviews generally focus on the hydrological, biogeochemical, and/or biological functions of these networked watershed components (for example, reviews on biogeochemical flux rates and storages within non-floodplain wetlands, Marton and others 2015). The novel contribution of this review is on mechanistically linking the hydrologically dynamic and biogeochemically reactive aspects of vulnerable waters—collecting, storing, transforming, and releasing water and entrained (that is, dissolved/particulate) materials—with the concepts of maintaining watershed states and imparting watershed resilience.

To develop these linkages, we first briefly review the scientific literature describing the mechanisms by which vulnerable waters affect watershed resilience. Below, we describe the functional contributions of vulnerable waters affecting watershed states and imparting watershed resilience focusing on (1) vulnerable-water extent and abundance, (2) hydrological functioning, and (3) biogeochemical functioning. For clarity, each review subsection is separated into reviewing and synthesizing the scientific literature on headwater streams, followed by non-floodplain wetlands.

### Known Extent and Proportional Abundance

**Figure 3.**
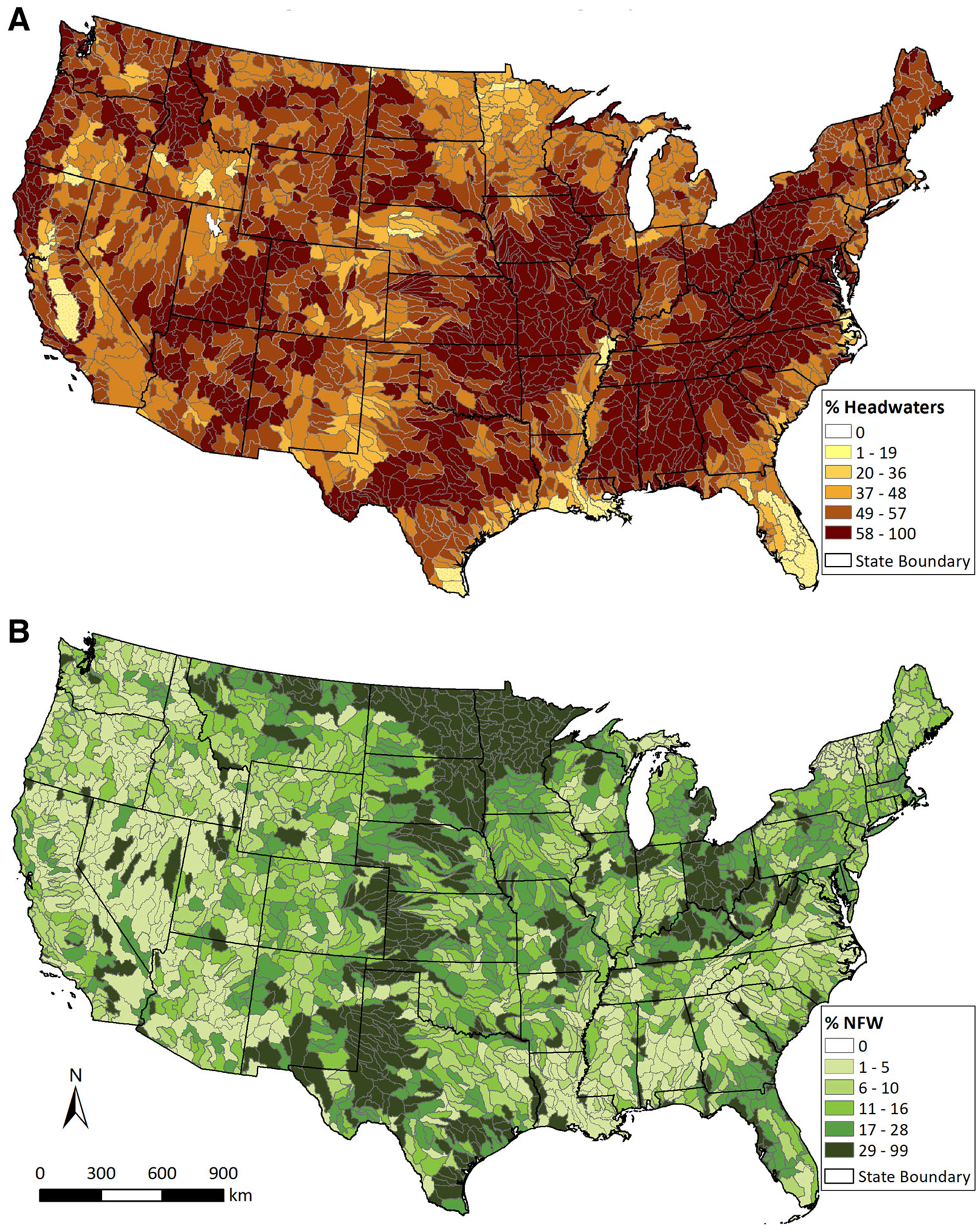
**A** and **B** Extent of mapped vulnerable waters in the conterminous USA, separately identifying the reported abundance within eight-digit Hydrologic Unit Code (HUC) watersheds for **A** Vulnerable Lotic Systems: 53% of the conterminous US stream length, or ~ 2,900,000 km, are headwater streams (defined as first-order streams using 1:100,000 data; Nadeau and Rains 2007, used by permission), 50% of which (~1,460,000 km) are reported to be intermittent or ephemerally flowing systems, and **B** Vulnerable Lentic Systems: non-floodplain wetlands (NFW, also known as geographically isolated wetlands, following Lane and D’Amico 2016; see Supplemental Material), approximately 23% of the area of freshwater wetlands in the conterminous USA was classified as non-floodplain wetlands, though wetlands smaller than 0.2 ha are typically unmapped.

Vulnerable waters consist of the most distal extent of the lotic systems (Wohl 2017) and non-floodplain wetlands (Lane and others 2018), including depressional wetlands and similar lentic systems embedded within uplands (Mushet and others 2015). These aquatic systems are often unmapped and have limited protection (Creed and others 2017; Mihelcic and Rains 2020). However, they are abundant and networked watershed components (Figure 3).

**Figure 4.**
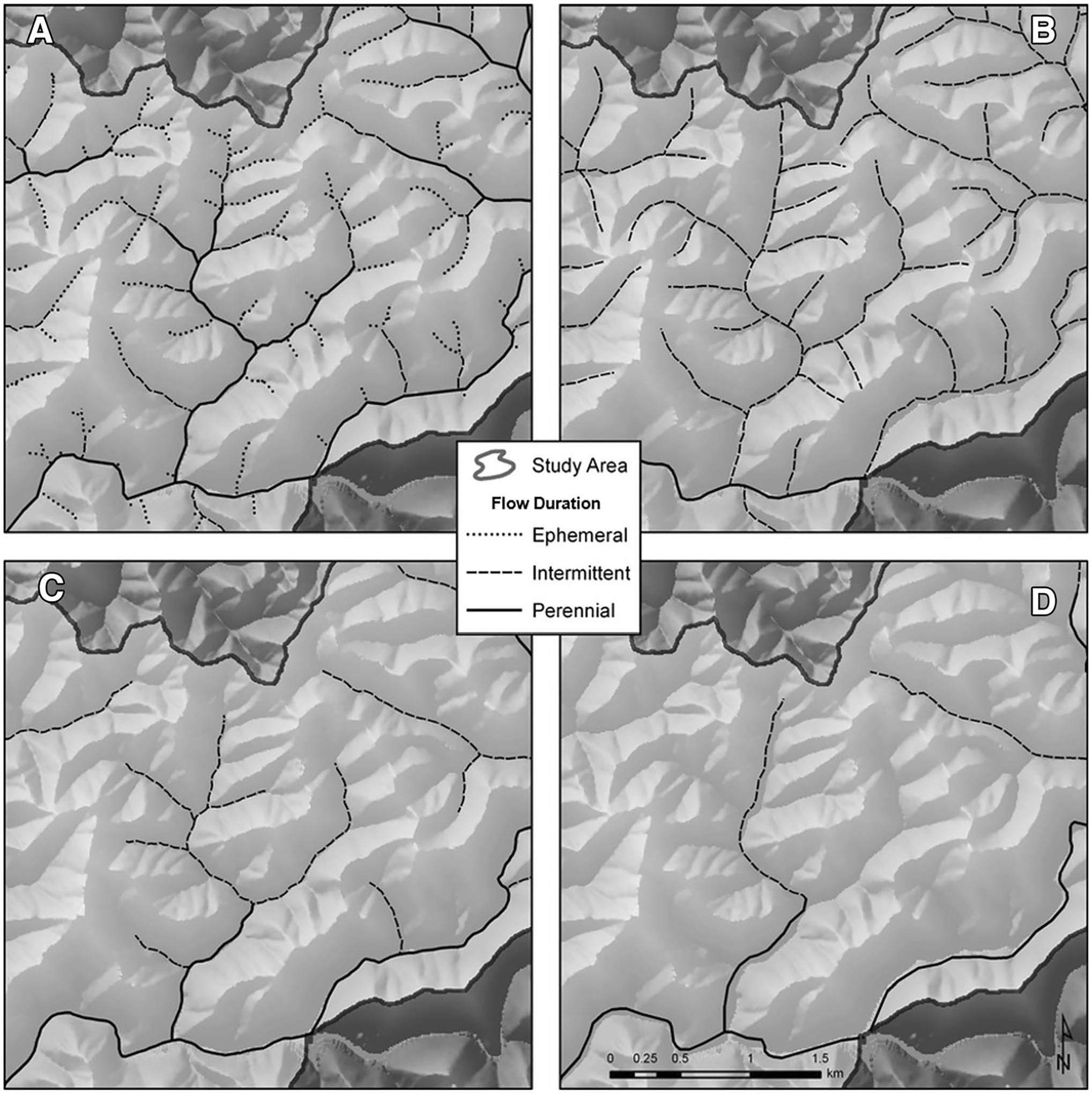
**A**–**D** Determinations of stream reach extent are affected by difficulties in accurately mapping narrow stream reaches and fluvial systems dominated by longitudinally dynamic ephemeral and intermittent flows. Fritz and others (2013, used with permission) contrasted **A** field-based efforts identifying stream origins across nine forested watersheds with **B** high-resolution mapped stream data from National Resources Conservation Service Soil Map (1:15,548 scale), **C** High-Resolution National Hydrography Dataset (NHD) Flowlines (1:24,000 scale), and **D** Medium-Resolution NHD Flowline (1:100,000 scale). Mapped stream extent decreased with increasing grain size.

Horton (1945) established that headwater streams, low-order systems inclusive of streams from coastal plains to mountainous physiographic regions, are the most abundant components of the fluvial network. Recent estimates suggest that nearly 89% of global longitudinal stream extent is comprised of these vulnerable waters (Allen and others 2018). In the USA, headwater streams represent approximately 50–80% of the total *currently mapped* conterminous US stream length (Nadeau and Rains 2007; Colvin and others 2019; Figure 3A), certainly an underestimate of headwater stream abundance (Hafen and others 2020; Fesenmyer and others 2021). Fesenmyer and others (2021) recently coupled nationally available high-resolution geospatial data with a contributing area threshold model, concluding that 48% of the conterminous stream length is likely ephemeral (43–56%, depending on flow area characteristics). Allen and others (2018) estimated that headwater streams are narrow (mean cross-sectional width of 32 cm ± 7 cm), which confounds the ability to accurately map their geospatial location and extent (Lang and others 2012; Vanderhoof and Lane 2019; Figure 4). Furthermore, flow can be highly variable in headwater streams, which affects estimates of vulnerable-water extent (Fritz and others 2013; Jaeger and others 2019; Zimmer and others 2020; Hammond and others 2021; Messager and others 2021).

In contrast to headwater streams, no global data are yet available on the potential extent of non-floodplain wetlands, representing a significant data gap in the effective management of these vulnerable waters (see, though, Borja and others 2020). freshwater–wetland areal extent (Lane and D’Amico 2016; this study, see Analyses in spatially data-rich areas such as the conterminous USA suggest that non-floodplain wetlands comprise approximately 16–23% of existing total Supplemental Material and Figure 3B). However, global wetland losses to date have been substantive; the USA alone has lost 50% of wetlands since European settlement, with some states having lost more than 90% (Dahl 1990). Recent data suggest wetland destruction continues with greater than 30% global areal losses since 1970 (Dixon and others 2016). Like streams, estimates of wetland distribution are hampered by the confounding effects of small areal extent (that is, wetlands < 1.0 ha, Cohen and others 2016), shallow depths, short hydroperiods (Wu 2018), overstory vegetation blocking extent delineation (Tiner and others 2015), and land-use change (Tiner 1997). In contrast to larger wetland systems, these small areal extent wetlands are disproportionately at risk of being altered or destroyed (Van Meter and Basu 2015; Serran and others 2017).

### Hydrological Functions

Headwater streams supply the majority of flow in most river systems (Alexander and others 2007; Fritz and others 2018). By providing flows to higher-order systems, headwaters directly affect watershed hydrological state and maintain resilience (for example, to drought perturbations). Even ephemeral or intermittent headwater streams without apparent surface flow are important for watershed resilience as they often have complex and abundant hyporheic flow that maintains the hydrological stability of down-gradient systems (Stanley and others 1997; Ebersole and others 2015; Covino 2017; Magliozzi and others 2018).

**Figure 5.**
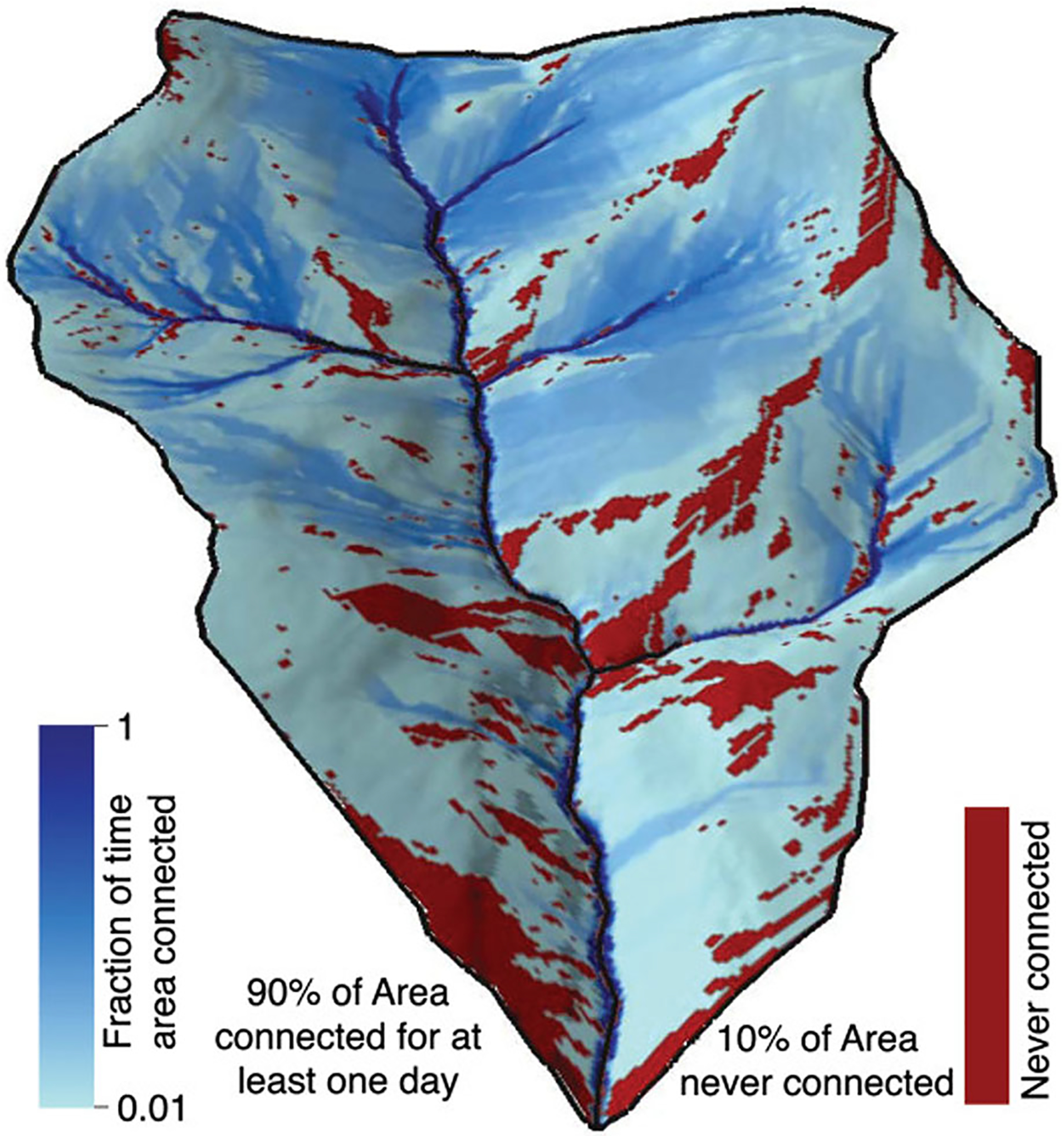
Flowing water within headwater streams reflects hillslope and drainage area connections that transmit dissolved constituents and particulate matter down-gradient through space and time, as demonstrated by the headwater connectivity map of Nippgen and others (2015; used by permission).

Flow from headwater streams maintaining down-gradient systems and imparting resilience is neither spatially nor temporally invariant but dynamic across watersheds as headwaters expand, contract, fragment, and reconnect across predictable spatial and temporal scales (Hewlett and Nutter 1970; Godsey and Kirchner 2014; Price and others 2021; Shanafield and others 2021). The heterogeneity of dynamic flow paths throughout a watershed’s headwater network creates storage and (subsequent) flow asynchronies (Saco and Kumar 2002; Moore and others 2015), which widen watershed latitude and deepen watershed resistance to hydrological disturbances by delaying and attenuating down-gradient storm flows and maintaining base flows (see Figure 1; Li 2019; Rupp and others 2021). Similarly, the spatial heterogeneity and temporal variability in the source area expansion and contraction of vulnerable waters (that is, watershed components generating overland flow, Figure 5) produce variability in the timing of headwater flow contributions at the reach scale (Jencso and others 2009; McGuire and McDonnell 2010; Klaus and others 2015; Bergstrom and others 2016). Flow contributed by headwater streams to down-gradient receiving waters is thus asynchronously integrated over time and space to maintain an adaptive and resilient down-gradient watershed state (for example, Chezik and others 2017; Rupp and others 2021).

Like their headwater stream, vulnerable water counterparts, non-floodplain wetlands are the flow origins of many watersheds, conveying watershed resilience by generating flow maintaining numerous down-gradient systems (Meyer and others 2003; White and Crisman 2016; Brooks and others 2018; Lane and others 2018; Thorslund and others 2018). For instance, in a chloride-tracer study across 260 North American catchments, Thorslund and others (2018) determined that non-floodplain wetlands on average generate runoff at 120% of the mean catchment rate (that is, they were disproportionately sources of down-gradient stream-flow) and up to 211% of the mean catchment rate in some circumstances. Similarly, non-floodplain wetlands of California’s Central Valley collected and contributed surface water down-gradient, often for months, and were part of an integrated and hydrologically dynamic headwater drainage network (Rains and others 2006).

Non-floodplain wetlands can also serve as focal areas for groundwater recharge, with some estimates of groundwater recharge through non-floodplain wetlands at greater than 300% of basin-wide averages (Rains 2011). Groundwater recharge from non-floodplain wetlands can then discharge down-gradient to maintain stream base flow (Thorslund and others 2018; Neff and others 2020), imparting greater watershed latitude and decreasing the probable impact and severity of hydrological disturbances modifying watershed state (for example, drought). For instance, Ameli and Creed (2017) modeled non-floodplain wetland interactions with drainage networks in Alberta, Canada, and found quantifiable contributions from non-floodplain wetlands occurred up to 30-km from the stream.

In contrast to flow generation and baseflow maintenance, non-floodplain wetlands can also act as flow-dampening systems, attenuating storm flow through storage functions and thereby providing watershed-scale resilience to hydrological disturbances (for example, deluge; Rains and others 2016). The watershed-scale resilience to hydrological disturbances provided by disconnected non-floodplain wetlands is demonstrated throughout the literature (see, for example, Lane and others 2018). For instance, Shaw and others (2012) noted 61% of a studied watershed’s wetlands were disconnected from overland flow paths, thereby performing watershed-scale storage functions dampening stream flow (see also Leibowitz and others 2016). Modeled hydrological retention decreasing stream peak flows by non-floodplain wetlands was similarly found by Fossey and Rousseau (2016) and Evenson and others (2018). Ameli and Creed (2019a) reported wetlands closer to streams performed greater peak flow attenuation than distal non-floodplain wetlands, while both types regulated base flow (that is, dampened baseflow variance; see also Shook and others 2021). Non-floodplain wetlands were likened to a hydraulic capacitor by McLaughlin and others (2014), providing watershed resilience to disturbance by modulating surficial aquifer variation and buffering stream base flow; non-floodplain wetlands functioned as groundwater sinks during wet periods and water sources during drier periods. The considered use of the term *hydraulic* by McLaughlin and others (2014, p. 7165) was to “…emphasize that the role these [non-floodplain wetlands] play in buffering surficial dynamics and downstream base flow is realized even where water in these systems may never physically reach downstream systems.”

### Biogeochemical Functions

Vulnerable waters are dynamic biogeochemical reactors within hydrological networks, transforming and sequestering materials and thereby affecting down-gradient physical and chemical characteristics and watershed state (Sanford and others 2007; Battin and others 2008; Larned and others 2010; Creed and others 2015; Hotchkiss and others 2015; Marton and others 2015; Cohen and others 2016; Cheng and Basu 2017; Fritz and others 2018; Arce and others 2019; Golden and others 2019; Gómez-Gener and others 2021). The spatial and temporal hydrological variability of vulnerable waters (for example, wetting up, drying down, pooling, connecting to other network components through surface and/or groundwaters, etc.) noted above controls redoximorphic-mediated reactions and microbial metabolism, affecting the delivery, timing, and concentrations of entrained materials moving into down-gradient waterways (for example, Enanga and others 2017; Senar and others 2018; Lynch and others 2019). The convolution of time-variant entrained material flows, high microbial activity, and physical assimilation, sequestration, and transformation rates within vulnerable waters mitigates watershed-scale biogeochemical disturbances (see Figure 2).

Headwater streams function as sinks, transformers, and pulsed sources of carbon, nitrogen, dissolved organic matter, sediment, other materials, and energy important to maintaining watershed states (Naiman and Sedell 1979; Minshall and others 1983; Holmes and others 1996; Benda and others 2004; Fisher and others 2004; Creed and Beall 2009; Larned and others 2010; Phillips and others 2011; Wohl and others 2012; Creed and others 2015; Enanga and others 2017; Senar and others 2018; Lynch and others 2019). For instance, research has shown that headwater systems can readily remove nitrogen (for example, Cooper 1990; Ranalli and Macalady 2010; Schmadel and others 2019). To wit, Scanlon and others (2010) reported that the abundance of biogeochemically reactive headwater streams in their study meant that they dominated watershed-scale nitrogen removal. Headwater streams in a Colorado study were estimated to constitute less than 25% of river length yet stored nearly 75% of the carbon via the sink functions of floodplain sedimentation and coarse wood deposition (Wohl and others 2012). Zierholz and others (2001) noted that headwater riparian wetlands stored greater than 20 years of nutrient and carbon-rich, annual, sediment yield, suggesting they store or process more material than they export (for example, Hotchkiss and others 2015).

Biogeochemical activity in headwater streams is variable in space (for example, along longitudinal gradients of ephemerally, intermittently, and perennially flowing systems) as well as through time (as given reaches may wet-up, create pools, and subsequently dry). Thus, biogeochemical transformation, sink, and storage potentials vary with flow along headwater stream networks as redoximorphic changes occur concomitantly with hydrological transitions between flowing, non-flowing (that is, pool formation stage), and dry reach conditions (Larned and others 2010; Lynch and others 2019; Gómez-Gener and others 2021). Precipitation-based rewetting events are periods of active biochemical processing in intermittent and ephemeral headwater streams, facilitated by pulses of novel terrestrial (allochthonous) material (Arce and others 2019). Subsequently, decreased flows can create standing pools with redox conditions conducive to further microbial activity (Hotchkiss and others 2015; Magliozzi and others 2018).

The dynamic biogeochemical reactivity and watershed-scale effects of ephemeral, intermittent, and perennial headwater streams are aptly described by the meta-ecosystems concept. Meta-ecosystems, introduced by Battin and others (2008), are spatially connected ecosystems where materials are sequestered and transformed through multiple abiotic and biotic processes along longitudinal gradients (Hedin and others 1998; Bernhardt and others 2005; Meixner and Fenn 2004; Kellman 2004; Fritz and others 2018). Within the meta-ecosystem concept, Battin and others (2008), as well as Hotchkiss and others (2015), described organismal metabolic rates (for example, microbial uptake velocities) as highest in headwaters (see also Peterson and others 2001; Alexander and others 2007). Furthermore, down-gradient portions of the flowing water network in the meta-ecosystem construct are redundantly structured to capture unconsumed material and utilize energy from upgradient sources (as well as novel material introduced to the network). Therefore, functional redundancies along longitudinal gradients that exist in the abundant headwater streams provide multiple opportunities for material transformation by different ecosystem components (that is, ephemeral, intermittent, and perennial stream reaches; see Battin and others 2008). The wide spatial distribution of headwater types within watersheds and the varied climatic, vegetative, and geophysical controls affecting the timing of flow (and flow permanence) provides watershed resiliency to biogeochemically processed pollutant disturbances (for example, Lynch and others 2019).

Like headwaters, non-floodplain wetlands are bioreactors (sensu Marton and others 2015) existing along a down-gradient connectivity continuum from highly connected to highly disconnected systems (Cohen and others 2016; Mengistu and others 2020). The important biogeochemical functions performed by these vulnerable waters affecting watershed state and resilience characteristics are increasingly well supported in the literature (Bernal and Mitsch 2013; Biggs and others 2017; Cheng and Basu 2017; Creed and others 2017; Lane and others 2018; Leibowitz and others 2018; Golden and others 2019). Similar to headwater streams, non-floodplain wetland biogeochemical functions emerge from the convolution of aerobic and anaerobic microbial processes, physical processes (for example, settling, photo-degradation), and residence time. In fact, drying events along headwater stream networks create conditions for now-isolated, low-order stream reaches to function similarly to ponded or perched wetland systems (for example, Rains and others 2006; Arce and others 2019).

Marton and others (2015) reviewed the scientific literature, estimating that non-floodplain wetlands sequestered or processed 21–317 g carbon m^−2^ y^−1^, 0.01–5.0 g phosphorus m^−2^ y^−1^, and 0.8–2.0 g nitrogen m^−2^ y^−1^ and found that residence time for microbial reactivity was an important rate-defining factor. Evenson and others (2018) modeled wetland water residence times at the watershed scale, noting a 75% decrease in residence time when smaller non-floodplain wetlands were removed from the landscape, resulting in lost opportunities for biogeochemical processing. In a synthesis of over 600 articles, Cheng and Basu (2017) determined that the first-order reaction rate constants for nitrogen and phosphorus were inversely proportional to wetland water residence times, a result that implies that > 50% of the nitrogen removal across all water bodies occurs in small wetlands (< 325 m^2^). Cohen and others (2016, p. 1980) found that most non-floodplain wetlands were “unambiguously small,” suggesting an outsized role in nutrient dynamics affecting watershed state (see also Golden and others 2019).

**Figure 6.**
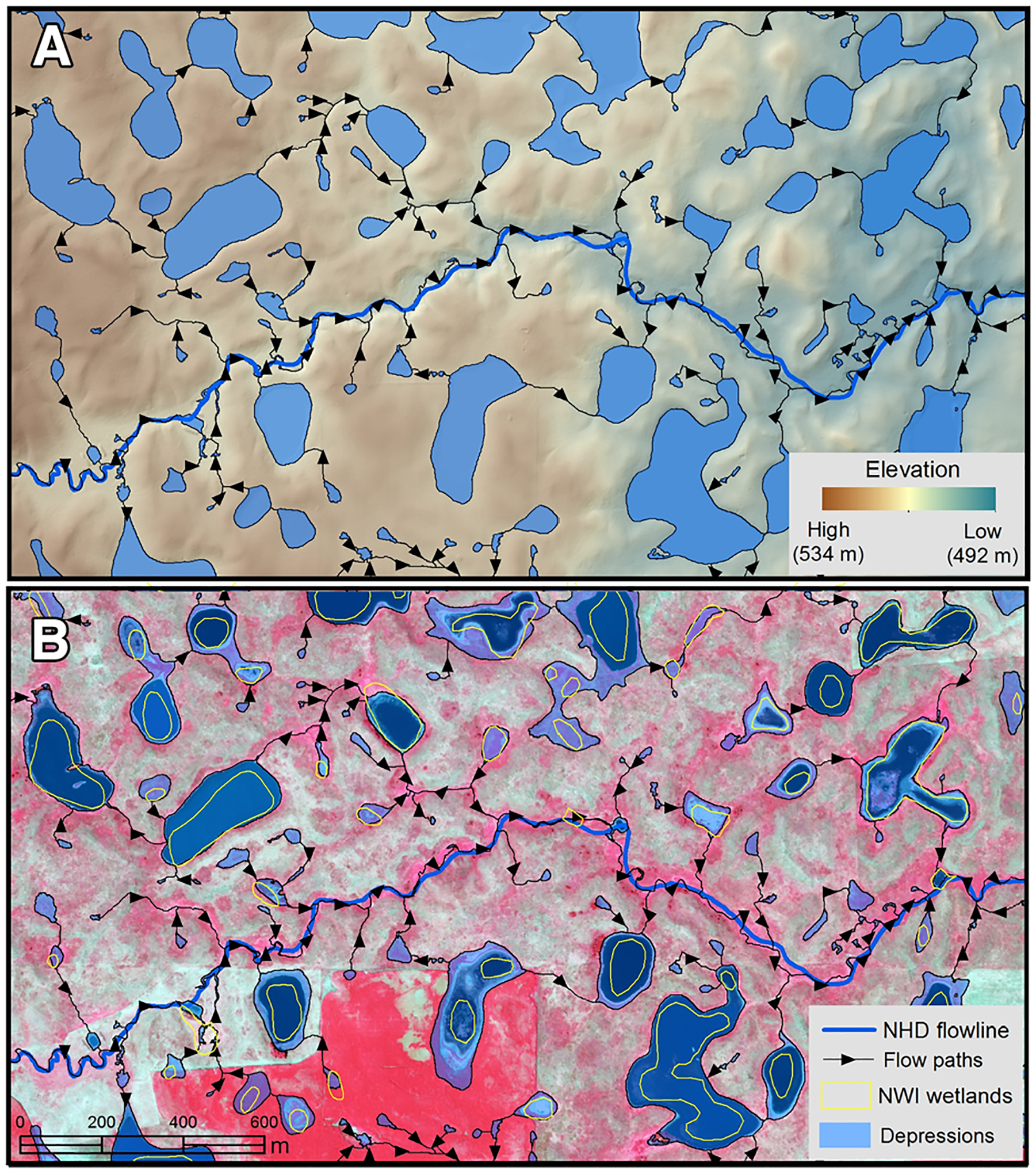
**A** and **B** Widespread spatial heterogeneity and climatic, volumetric, and geophysical characteristics control non-floodplain wetland biogeochemical (and hydrological) functions affecting watershed resilience. Wu and Lane (2017) **A** identified potential wetland depressions and connectivity flow paths to a National Hydrography Dataset (NHD) river in a North American watershed (Pipestem River, North Dakota, USA) using lidar; the variability in wetland size, estimated volume, perimeter to area (Cohen and others 2016) and bathymetric properties (Cheng and Basu 2017) were found by Evenson and others (2018) to affect biogeochemical and hydrological functions. In addition, Wu and Lane (2017) **B** contrasted lidar-based non-floodplain wetland depressions with the best available National Wetland Inventory (NWI) data, demonstrating both regional wetland expansion since the baseline aerial imagery were acquired, as well as limitations to remotely identifying non-floodplain wetland systems.

The ameliorating effects of non-floodplain wetlands on watershed-scale biogeochemical disturbances, such as excessive nitrogen, emerge from the cumulative contributions of the non-floodplain wetlands across the landscape (Evenson and others 2018; Golden and others 2019). Much like the aforementioned meta-ecosystem concept applied to headwater stream networks, (Battin and others 2008), non-floodplain wetlands can similarly be considered to exist as a series of highly connected to (at times) highly “disconnected” bio-reactive ecosystem components interacting within watersheds (Leibowitz 2003; Marton and others 2015; Rains and others 2016). Individually, non-floodplain wetlands consolidate flows, intersect flow paths, and provide residence time and biophysical conditions for microbial activities, then release waters through surface, near-surface, atmospheric, or deep groundwater recharge (Rains 2011; Rains and others 2016; Neff and Rosenberry 2018; Neff and others 2020). Cumulatively, non-floodplain wetlands contribute to watershed-scale resilience due to their widespread spatial heterogeneity—even within individual watersheds—imparting variability in factors affecting biogeochemical reactivity or residence time (such as size, depth, and volume), and down-gradient flow paths (for example, Figure 6). Watershed resilience to biogeochemical disturbances thus emerges from the functional redundancies of many non-floodplain wetlands within a watershed assimilating (storing, transforming) biogeochemical material at different rates, then transiting the (remaining) entrained or dissolved materials and energy down-gradient for subsequent processing along reactive flow paths (Mengistu and others 2020) or to other aquatic components.

## Disturbances TO Vulnerable Waters Decrease Watershed Resilience

Due to their state-defining functions affecting the storage, flux, transformation, and conveyance of water and entrained solutes and particulates, it is evident that vulnerable waters provide watershed-scale hydrological and biogeochemical resilience. The magnitude of the influence of vulnerable waters on watershed state is correlated with their cumulative abundance, functional redundancy, and exposure to state-defining hydrological and biogeochemical inputs (for example, Creed and others 2003; Creed and Beall 2009; McLaughlin and others 2014; Mengistu and others 2014). Thus, vulnerable waters within some watersheds, such as those with a low headwater drainage density or limited areal extent of non-floodplain wetlands, may have a relatively minor influence on the magnitude, duration, frequency, or intensity of state-defining hydrological or biogeochemical effects (for example, Sanford and others 2007; Ali and English 2019). Similarly, the influence of vulnerable waters in maintaining resilience to disturbances may wane with increasing watershed area and the concomitant volumetric mixing and dilution effects (for example, Benda and others 2004; Kellman 2004; Covino 2017; Rajib and others 2020a), or be moderated based on terrestrial drainage properties (for example, Klaus and others 2015; Neff and others 2020).

However, the disturbance-driven marginal loss of extant vulnerable waters and their functioning that may occur with filling, ditching, armoring, channelizing, water abstraction, and climate change effects cumulatively alters the response, recovery, and reorganization of watershed-scale hydrological and biogeochemical states. In other words, the resilience of a watershed’s state (that is, latitude and resistance) is weakened by the cumulative loss of vulnerable waters, which marginalizes and discounts the suite of functions, feedbacks, and variance-dampening effects provided by vulnerable waters. Thus, incremental loss or hydrological modification of vulnerable waters can have increasingly pronounced cumulative effects, decreasing system resilience to disturbances and leading to a state-changing regime shift (for example, McKenna and others 2017). For example, decoupling non-floodplain wetland-mediated nutrient assimilation and removal via ditching and drainage and both deepening and straightening headwaters decreases travel times and increases nutrient and sediment loading to down-gradient aquatic systems (Golden and others 2019). Foufoula-Georgiou and others (2015) noted increased drainage intensity and tiling accompanying land-use change altered mean annual stream flows threefold in a Minnesota (USA) subbasin, increasing sediment loads to the aquatic network and precipitating a possible regime shift to a high-flow, sediment-laden system. Evenson and others (2018) demonstrated that destruction of vulnerable waters (non-floodplain wetlands) would increase the frequency and magnitude of flood events in an 1800-km^2^ Midwestern United States watershed; flooding events are purveyors of regime shifts in aquatic systems (Robinson and Uehlinger 2008; Dodds and others 2010). Conversely, Jones and others (2018) reported that watershed-scale hydrological storage capacity across multiple watersheds within a 14,000-km^2^ Mid-Atlantic Coastal Plain (USA) region could be increased by 80% through simply plugging ditched and drained vulnerable waters (that is, depressional non-floodplain wetlands), increasing watershed resilience to drought and flooding events by providing stream baseflow maintenance, stormflow and the potential flood event desynchronization, and mediating both nutrient and sediment flux to down-gradient systems (for example, Chesapeake Bay, USA).

It is evident that accounting for disturbances to vulnerable-water hydrology is crucial to understanding both the hydrological *and* biogeochemical effects of vulnerable waters on watershed resilience (Alexander and others 2009; Palmer and others 2010). In other words, replumbing watershed hydrology, which alters hydrological storages and fluxes from the distal and typically abundant vulnerable waters, can dramatically affect watershed-scale hydrology and concomitantly watershed-scale biogeochemistry (Marton and others 2014, 2015; Cohen and others 2016; Rains and others 2016; Fritz and others 2018). For instance, climate change has increased precipitation affecting the Prairie Pothole Region (PPR, an 800,000 km^2^ area of the Midwestern United States and southern Canada) resulting in the hydrological expansion and merging of depressional non-floodplain wetlands (McCauley and others 2015; McKenna and others 2017) and the dramatic expansion of terminal lake systems. Devils Lake, a large terminal lake in the PPR, expanded from 180 to 695 km^2^ between 1992 and 2013, increasing more than 9 m in lake depth (Todhunter 2018). New regional precipitation patterns have necessitated increased terrestrial tiling to facilitate landscape drainage. Changes in both precipitation patterning and increased landscape drainage through tiling have in turn resulted in increased concentrations of total dissolved solutes (TDS, salts) within vulnerable waters (LaBaugh and others 2016), increased discharge within headwater streams and rivers, and increased water permanence on the landscape (Vanderhoof and others 2018), also affecting vulnerable water microbial activity (for example, through potential redox changes, Zeng and others 2011). Mushet and others (2020) and McKenna and others (2017) concluded climate change altered precipitation coupled with hydrological modifications (for example, tile drainage, consolidation drainage of smaller vulnerable waters [wetlands]) within watersheds of the PPR are presaging a region-wide ecohydrological regime shift to watersheds with deeper ponded waters and altered TDS concentrations in wetlands, larger and more permanent and deeper lakes, and greater stream and river discharge.

A further, similarly dramatic indicator of the coupled effects of vulnerable-water hydrological and biogeochemical alterations on watershed resilience and system steady state is found in areas of mountaintop mining, wherein waste rock from surface mines is disposed of in headwater stream valleys (Palmer and others 2010), which fundamentally alters down-gradient system hydrology (for example, flow permanence; Ferrari and others 2009; Bernhardt and Palmer 2011). Hydrological changes from compaction and valley fill concomitantly alter headwater stream functional biogeochemistry (for example, changing redoximorphic gradients due to changing flow permanence, Lindberg and others 2011; Ross and others 2016; Gómez-Gener and others 2021). In addition, these dramatic changes also physically alter the terrestrial contributing area, which frequently, if not typically, transition the system to an alternative hydrological and biogeochemical (and biological; Petty and others 2010) steady state.

**Figure 7.**
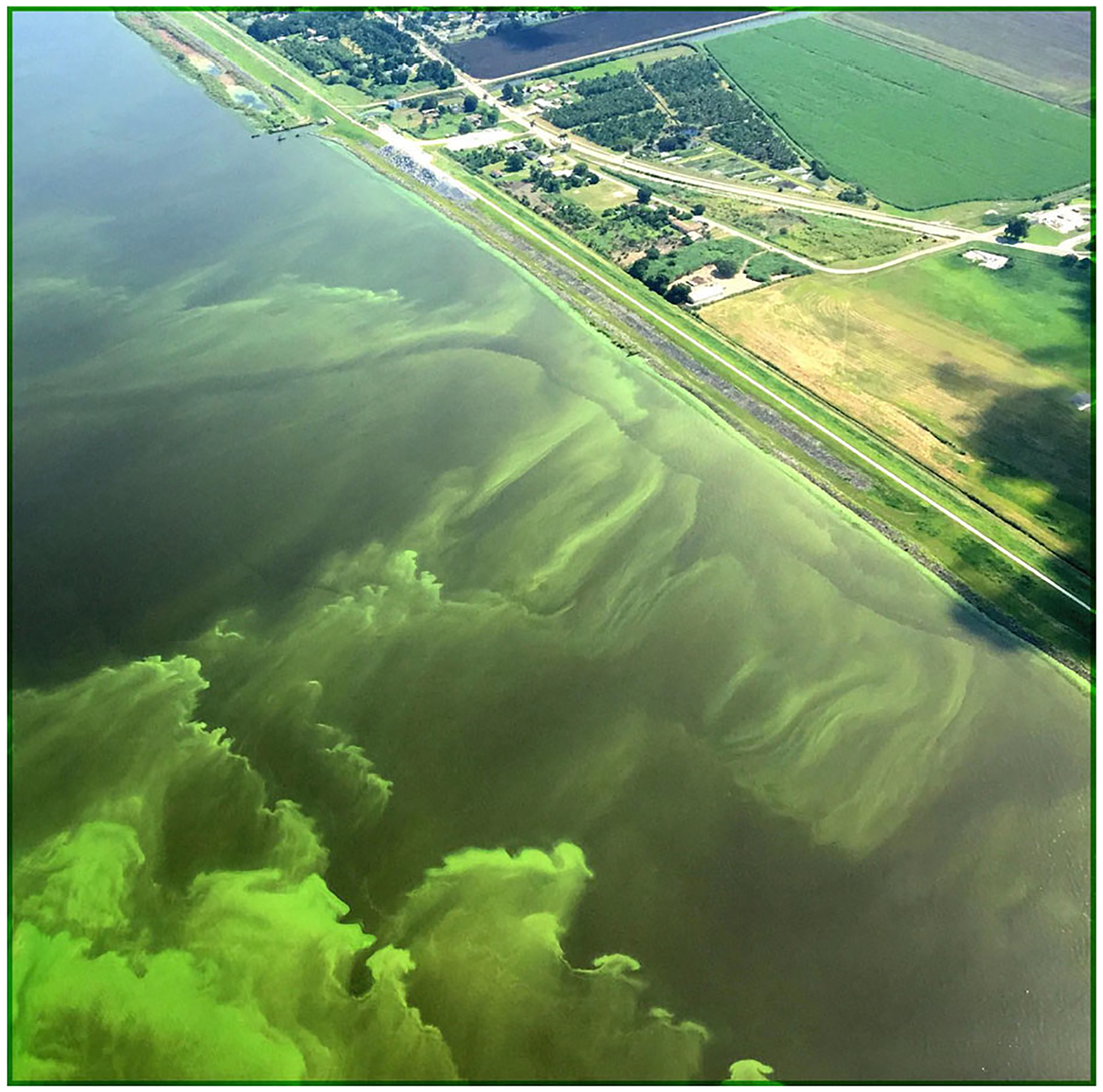
Lake Okeechobee (Florida, USA), the terminus of several significantly altered watersheds, has transitioned from a stable oligotrophic to stable eutrophic lake system with recurrent algal blooms (see state transitions in Figure 2). Current management efforts are focusing on mitigating disturbances to upgradient non-floodplain wetlands and their attendant hydrology, increasing watershed-scale surface water storage to capture overland flow and facilitate sedimentation, pollutant sequestration, and nutrient assimilation, attempting to limit the resilience of the existing eutrophic state (Zhang and others 2009). Image source: USGS 2016, image in the public domain (https://www.usgs.gov/media/images/algal-bloom-lake-okeechobee-florida-2016 , acquired December 2020).

As noted, watershed changes to alternative states (that is, regime shifts, sensu Lant and others 2008) occur when watershed resilience in a given state is overwhelmed (Walker and others 2004; Hayashi and others 2016), disturbance thresholds are crossed, and existing states undergo a transformational change (Scheffer and others 2012; Ratajczak and others 2018; see Figure 2). Once transitioned, the resilience of the new state may be substantial, making it difficult to revert to the previous or another new state (Botkin and Sobel 1975; Beisner and others 2003; Angeler and Allen 2016; Falkenmark and others 2019). For instance, once a lake system has transitioned from oligotrophic to eutrophic it may be energetically, technologically, economically, or politically infeasible to remove the continually resuspended sediments maintaining the lake in a eutrophic state. As an example, decades of agricultural development within the watershed that drains into Lake Okeechobee, Florida, resulted in ditched and drained non-floodplain wetlands and both straightened and shortened headwater streams to facilitate and expedite watershed drainage. Consequently, rather than being sequestered or transformed in the watershed’s vulnerable-water network, pollutants became entrained down-gradient and ultimately entered Lake Okeechobee, which transitioned from a stable oligotrophic to a stable eutrophic system state. While the system has already transitioned to this new state, management efforts are now focusing on limiting the resilience of the *current* system state (for example, that of a eutrophic system, Figure 7) by restoring upgradient non-floodplain wetlands to decrease watershed-scale nutrient loading and facilitate sedimentation, sequestration, and nutrient assimilation (Zhang and others 2009). Hence, eutrophication management within a watershed drainage network can focus on vulnerable waters as solutions (for example, Zhang and others 2009; Yang and others 2010; Ali and English 2019), while also considering nutrient legacy effects in lake sediments (Ostrofsky and Marbach 2019). These findings invite future research that tests similar hypotheses regarding large river, floodplain, and estuary state changes resulting from upgradient modifications of vulnerable waters.

## Maintaining Resilience in Watersheds: Principles for Management

It is evident that vulnerable waters within watersheds comprise a significant majority of hydrological networks (Horton 1945; Nadeau and Rains 2007; Allen and others 2018; see Figure 3) and a substantive proportion of wetland extents (for example, Lane and D’Amico 2016). Furthermore, it is clear that the presence of functioning vulnerable waters affects watershed state and improves watershed resilience to disturbance by providing substantial hydrological (for example, Rains and others 2016; Fritz and others 2018) and biogeochemical (for example, Creed and others 2015; Marton and others 2015) functions. These functions provide state-maintaining “negative” feedbacks (that is, deepening resistance to change), reduce pollutant concentrations, and dampen the magnitudes of fluxes to down-gradient systems by serving as flow consolidators and capacitors, bioreactors and asynchronous flow integrators (Saco and Kumar 2002; McLaughlin and others 2014; Marton and others 2015; Ali and English 2019).

**Figure 8.**
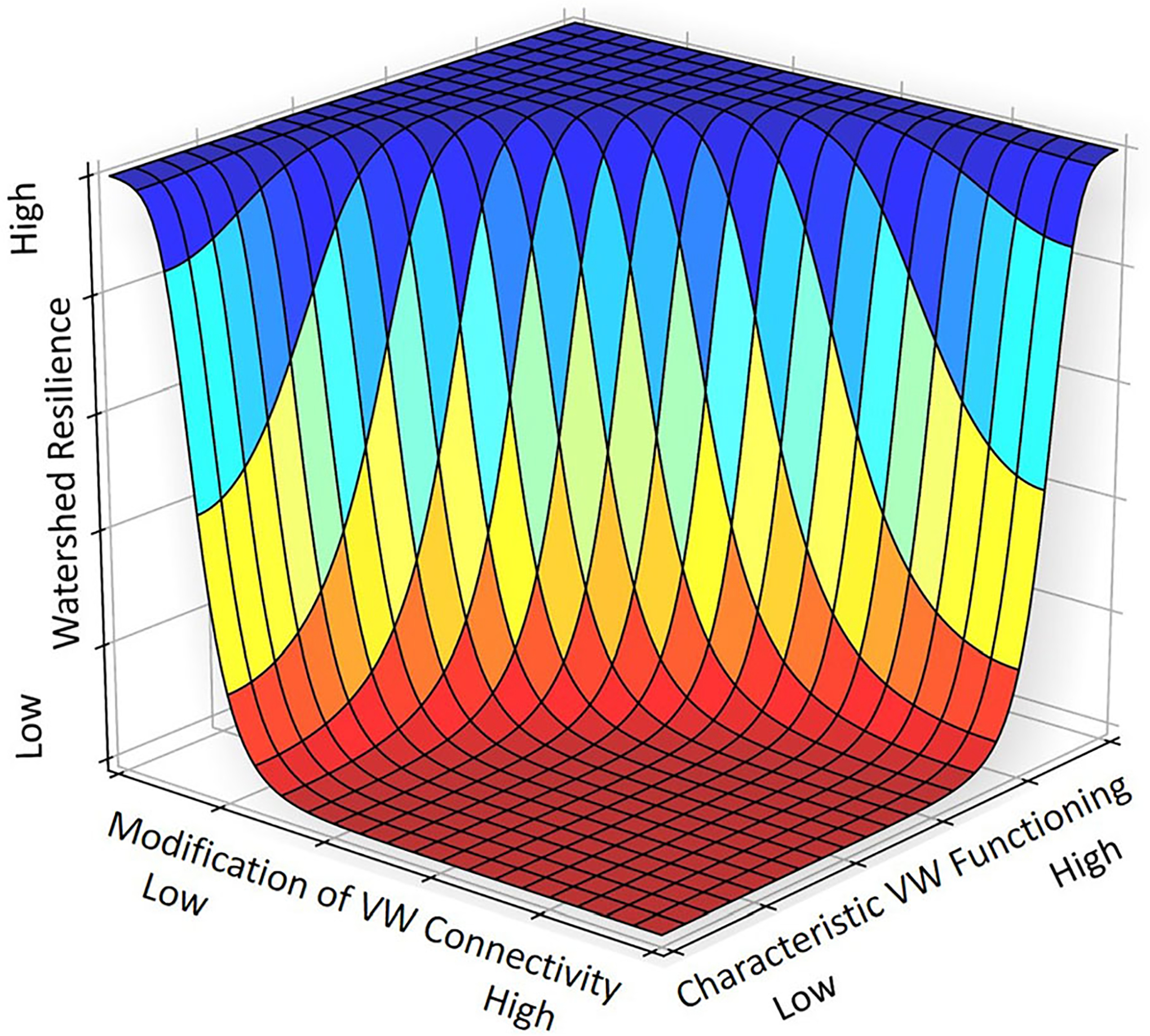
Modifications to vulnerable-water (VW) connectivity and isolation gradients and characteristic VW functioning affect watershed resilience to disturbances that modify current watershed state. Watershed-scale conceptualized relationships between anthropogenic modifications to VW networked connectivity/isolation gradients on the *y*-axis, characteristic watershed functions performed by VWs on the *x*-axis (for example, VW biogeochemical and hydrological flux-dampening functions as a proportion of watershed functions), and watershed resilience (*z*-axis). Existing watershed state and resilience (that is, ability to absorb disturbance and concurrently adapt while retaining equal function, structure and structural integrity, identity, and feedbacks), emerges from the convolution of unaltered VW flow path connectivity and isolation gradients and extant vulnerable-water functioning (that is, high characteristic VW functioning).

The hydrological and biogeochemical functions performed by vulnerable waters promote watershed-scale resilience that emerges from the interactions between interconnected vulnerable waters and the terrestrial landscapes they drain (for example, Battin and others 2008). Critically, it is the presence of direct and indirect connections and disconnections between networked components of the watershed that provide evidence for the role of vulnerable waters in watershed-scale hydrological and biogeochemical functioning, states, and resilience [Figure 8, for example, subsurface flows sensu (Covino 2017), hydraulic effects sensu (McLaughlin and others 2014), hydrological effects sensu (Mengistu and others 2020)]. These functions occur across a gradient from highly connected to disconnected vulnerable waters. Disconnections (that is, isolated conditions) such as stream network fragmentation and wetland perching often provide the necessary redoximorphic conditions for optimal vulnerable-water biogeochemical functioning, while concurrently optimizing hydrological storage/attenuation functions, thereby adding to watershed-scale resilience (for example, USEPA 2015; Cohen and others 2016; Skoulikidis and others 2017; Fritz and others 2018; Lane and others 2018; Schofield and others 2018; Gómez-Gener and others 2021).

As we have illustrated, watershed resilience emerges when time-varying hydrological and biogeochemical fluxes from the terrestrial landscape are conveyed to and through vulnerable waters wherein biogeochemical disturbances (for example, excessive nutrients) are typically processed, and hydrological disturbances (for example, excessive flows) are often attenuated. Using this context, we have identified four principles for consideration by natural resource managers that endeavor to effectively manage watersheds for a beneficial ecological state. The principles will support the sustainability of ecological, hydrological, and biogeochemical services emanating from vulnerable waters that are important to human health and well-being, economic development, resource production, and watershed resilience into the future (Falkenmark and others 2019). These principles are based on the emerging understanding of the importance of vulnerable waters to watershed management endpoints (for example, flood attenuation and nutrient reduction), as well as the novel coupling of resilience theory and vulnerable-water science for watershed management articulated here.

### Principle 1: Comprehensively Map the Dynamic Extent, Spatial Arrangement, Networked Connectivity, and Function of Vulnerable Waters

The cumulative interactions between extant vulnerable waters and their variable source and terrestrial drainage areas provide enhanced opportunities for hydrological and biogeochemical functioning, which can maintain and strengthen a watershed’s resilience to hydrological and biogeochemical disturbances. Modification and destruction of vulnerable waters changes watershed structure and alters watershed adaptability to anthropogenic disturbances, auguring hydrological and biogeochemical change to down-gradient system states. *The first principle acknowledges that watershed-scale functions provided by vulnerable waters emerge from the quantity, spatial arrangement, temporal variability, functional diversity, and dynamic networked connectivity of vulnerable waters, and watersheds depend on these properties to provide adaptability and resilience to disturbances*.

The functional redundancy of vulnerable waters performing similar functions along the full extent of the aquatic network affects hydrological and biogeochemical flux magnitudes at the watershed outlet (for example, Shaw and others 2012; McLaughlin and others 2014). The incremental watershed-scale loss of any one vulnerable water may be inconsequential. However, the cumulative loss of many vulnerable waters decreases the functional redundancy inherent in watersheds, thereby decreasing watershed resilience and likely affecting watershed management endpoints and goals (Nyström and Folke 2001; Cumming 2011; Allen and others 2016). This concept of watershed-scale functional redundancy in complex systems is similar to Ehrlich and Ehrlich’s (1981) species extinction metaphor of losing rivets in an airborne plane. Loss of a few vulnerable waters only marginally affects watershed state. However, the loss or modification of many vulnerable waters performing redundant hydrological and biogeochemical functions asynchronously networked within a watershed may perilously engender a regime shift. Thus, vulnerable water losses over time incrementally increase watershed precariousness, while the cumulative effects of many marginal losses concurrently decrease watershed latitude and resistance to disturbances, decreasing watershed resilience, and ultimately affecting a state change (that is, the plane suffers a catastrophic failure and subsequent “rapid uncontrolled disassembly,” Ratajczak and others 2018).

We agree with Angeler and Allen (2016, p. 628) who noted, “[t]he roles of within- and among-system connectivity are critical to understanding ecological regime shifts and, therefore, resilience.” Hence, practical application of the first principle requires not only knowing the location and extent of vulnerable waters but also knowledge of vulnerable water connectivity with and effects on other components of the watershed system (Battin and others 2008). In practice, repeated measurements with high temporal and spatial resolution geospatial data provide useful information for identifying and mapping the dynamics of temporally variable and spatially heterogeneous vulnerable waters (Wood and others 2011; Beven and Cloke 2012; Serran and Creed 2016; Wu 2018; Wu and others 2019a,b). These data can be integrated into empirical analyses or model simulations that quantify watershed component effects on measured system-defining variables, such as in-stream water flows (for example, Ameli and Creed 2019a,b) and water quality conditions (for example, Bellmore and others 2018; Hansen and others 2018; Mengistu and others 2020). Furthermore, integrated high-resolution data and modeling applications provide a meaningful representation of watershed-scale vulnerable-water restoration effects (Jones and others 2018). Where headwater stream and non-floodplain wetland restoration occurs within watersheds can greatly affect measurable outcomes (for example, Cheng and others 2020; Evenson and others 2021) and similarly affect watershed resilience to disturbance.

### Principle 2: Determine State-Changing Hydrological and Biogeochemical Thresholds

The loss of spatially heterogeneous vulnerable waters and their interactions with lands they drain affects resilience by decreasing system latitude and resistance and increasing system precariousness, transiting the system toward a regime shift (see Figure 2). A critically important management question thus emerges: what is the transition point or threshold beyond which the incremental loss of vulnerable waters instigates system instability auguring a potential state change? *The second principle articulates that the transition point or threshold beyond which a watershed departs the basin of attraction of one state and enters an alternative state should be determined and targeted for management* (Booth and Jackson 1997; Dodds and others 2010; van de Leemput and others 2015; Zelnik and Meron 2018).

Thresholds can be ecologically defined (Walker and others 2004). For instance, ecologically determined thresholds may be identified for flow regimes to protect ecological integrity (for example, maintenance of minimum river flows for the protection of aquatic organisms). Hydrological thresholds may also be identified for spatial connectivity (for example, McLaughlin and others 2019) or flood frequency (for example, maintenance of non-floodplain wetland storage and floodplain storage to reduce peak flows; Rajib and others 2020a). Although characteristics of different watershed states can be specifically defined, determining effective transition point(s) between states is much more problematic—though not insurmountable—due to the multiplicity of interacting drivers affecting states (see Principle 3; Sayer and others 2006; Hecky and others 2010).

The identification of these state transition thresholds remains key to facilitate adaptive management (see Principle 4, below) and limit unwelcome state transitions. The increasing availability of measured data and application of models at finer spatial grain sizes and higher temporal frequency provides opportunities for state and threshold identification (for example, Schindler and others 2016; Bernhardt and others 2018; Gleeson and others 2020). For example, recent work in flowing waters by Diamond and others (in press) identified both riverine state (for example, turbidity, nutrient concentration) and linked metabolic (that is, gross primary production, ecosystem respiration) regime shifts across thresholds, with implications as early warning indicators for river management. Studies in Europe and North America have identified phosphorus thresholds to limit lake eutrophication (Fastner and others 2016; Schindler and others 2016; see also Falkenmark and others 2019; Zipper and others 2020). Recent research linking the theory to application has proliferated across ecosystem types (Scheffer and others 2001; Folke and others 2004; Biggs and others 2009). For instance, early warning signals such as critical slowdowns (for example, Verbesselt and others 2016) and increases in measured variance (that is, flickering, see Figure 2) and autocorrelations have presaged regime shifts and allowed determination (Pace and others 2017) and, importantly, reversal (Wilkinson and others 2018) of transitions across state thresholds. Identifying and predicting thresholds allow for informed watershed management to change the trajectory of a transition to that of one for sustainable futures.

### Principle 3: Identify Drivers of Change and Prioritize Management Activities

Multiple interacting factors affect vulnerable waters and hence watershed state and resilience, from direct climate change-induced non-stationarity disturbance effects of temperature and precipitation on connectivity and functioning, to human activities that destroy vulnerable waters or increase pollutant and contaminant loading to watershed systems (for example, Uden and others 2015; McKenna and others 2017; Senar and others 2018; Vanderhoof and others 2020). However, robust data collection and interrogation, analysis, and synthesis can result in deducing the extent, duration, and intensity of the disturbance effects (that is, main drivers) affecting watershed states and resilience (for example, Hipsey and others 2015; Hansen and others 2018; Van Meter and others 2018). *The third principle therefore calls for identifying and characterizing the extent, duration, magnitude, and intensity of key disturbance drivers precipitating watershed change for prioritizing management*. This is crucial for managing for resilient watersheds because knowing the disturbance forces (for example, land-use conversion, modification of irrigation or tile drainage, grazing intensity, fire, insect outbreaks) that are “pushing” the watershed toward the alternative state transition point or threshold noted in Principle 2 (and see Figure 2) allows for prioritizing vulnerable-water-based management solutions and coupled socio-environmental strategies to mitigate risks associated with a loss of watershed resilience (Liu and others 2007).

For example, a watershed moving toward a threshold-transitioning alternative state is the Lake Winnipeg watershed in Canada. Here, nutrient-rich inputs from agricultural activities (that is, primary drivers of this change) are pushing this mesotrophic lake—the world’s 10th largest by surface area—to a eutrophic system, with recurrent algal blooms of such magnitude and frequency that it has been called both “Canada’s sickest lake” and “the most threatened lake in the world” (quoted within Ali and English 2019). The lake is likely on the verge of a regime shift, crossing a threshold from its historical mesotrophic state to a bi-stable or an alternative eutrophic state (Bunting and others 2016; Figure 2). Though other contributors to this change are still being researched, Ali and English (2019) recently underscored the co-occurrence of algal blooms (indicators of pulsed nutrient loading) with watershed-scale nutrient-enriched runoff through modified watershed drainage. Importantly, their analyses determined that modification of vulnerable-water connectivity in the watershed has been driving the production of major Lake Winnipeg algal blooms. This is corroborated by Yang and others (2010), who modeled an approximately 23% nutrient load reduction in a 250-km^2^ Lake Winnipeg-contributing watershed with vulnerable water restoration resulting in decreased peak discharge and sediment loads. In other words, the pulsed nutrient loading (feedbacks positively hastening a regime shift) that drives algal bloom occurrence can be potentially mitigated by a management focus on the protection and restoration of functioning headwater systems and non-floodplain wetlands, thereby decreasing watershed-scale nutrient loading by increasing hydrological residence time and biogeochemical processing in vulnerable waters.

Watershed-scale analyses can inform drivers of state change, though in application such analyses can be exceedingly complex (for example, Evans and others 2005; Roulet and Moore 2006; Wood and others 2011; Beven and Cloke 2012; Archfield and others 2016; Blum and others 2020). However, scientists can provide natural resource managers with information on potential drivers affecting watershed state to prioritize the application of limited human and financial resources to mitigate potentially undesirable watershed states or adapt to looming state changes (for example, Gannon and others 2013).

### Principle 4: Adaptively Manage Watersheds

In the three principles above, we articulated the inherent requirements of vulnerable-water management for adaptable and resilient watersheds. *The fourth principle, adaptive management, embodies the practical application of the preceding principles by allowing for data-driven management course changes to achieve goals. Adaptive watershed management encourages bold experimentation to find solutions; decisions responding to vulnerable-water characteristics and functioning should be informed by increasingly data-rich analyses and syntheses of temporally dense and high-spatial-resolution watershed-scale data*.

The fourth principle of adaptive watershed management is thus reliant in practice upon increases in spatial and temporal data collection, granularity, and analyses supporting the incorporation of vulnerable waters into quantification of hydrological and biogeochemical storages, transformations, and fluxes within and emanating from watersheds (for example, Ali and English 2019). Sufficiently armed with those data that more fully describe the physical reality and granularity of the landscape and interactions therein (for example, Rajib and others 2020b; Fesenmyer and others 2021; Golden and others 2021), effective experimentation and subsequent management can make midcourse corrections to improve watershed resilience and desirable outcomes (Pace and others 1999, 2017; Furniss and others 2010; Hoque and others 2012; Gannon and others 2013; Standish and others 2014; Garmestani and others 2020). For instance, Pace and others (2017) and Wilkinson and others (2018) identified early warning indicators of cyanobacterial blooms in experimentally manipulated lake systems; adaptively decreasing nutrient loads reversed the bloom extent.

## Scientific Needs Informing the Maintenance of Watershed Resilience

Despite scientific evidence of the importance of vulnerable waters to maintaining desirable watershed states (Creed and others 2017), most analyses attempting to quantify watershed hydrological or biogeochemical states *do not* incorporate vulnerable waters into their study frame (see Golden and others 2021). Indeed, though efforts are ongoing for inclusion of citizen science (for example, Seibert and others 2019) and a growing chorus of researchers noting the importance of collecting vulnerable water data for large spatial extent analyses (for example, Jaeger and others 2021), there remains a paucity of data on the location of vulnerable waters and the storage and fluxes within and from vulnerable waters affecting down-gradient systems. We therefore identified the following research needs associated with managing for resilient watersheds, providing increasingly available data to make the principles more fully actionable.

1. *Spatial Extent* Vulnerable waters comprise a majority of stream lengths (Allen and others 2018; Messager and others 2021) and a substantive proportional abundance of wetlands (for example, Lane and D’Amico 2016). However, accurately mapping the current and dynamic spatial extent of headwater streams and non-floodplain wetlands remains a pressing data need. Without these data, it is not possible to meaningfully manage vulnerable waters and their terrestrial interactions, identify critical vulnerable waters controlling fluxes or connections (for example, Larsen and others 2012; Uden and others 2014; Ali and English 2019), or effectively quantify watershed state-defining functions. With these data in hand, it may be possible to develop management scenarios incorporating past, present, and projected future vulnerable-water extents, conditions, and impacts on watershed state and resilience (for example, Rains and others 2013).
2. *Spatial Configuration* The local-scale effects of individual wetlands have been widely studied, yet the watershed-scale effects of wetland complexes have been less frequently considered (for example, Acreman and Holden 2013; Golden and others 2019; Klammler and others 2020), including the coupled groundwater–surface interactions of these vulnerable waters and those contributions to watershed-scale resilience (McLaughlin and others 2014; Neff and others 2020). Nevertheless, Cohen and others (2016) hypothesized that landscape functioning emerges from the convolution of the individual effects of all wetlands, both those directly abutting rivers and streams (for example, floodplain wetlands) and those in more remote locations (for example, non-floodplain wetlands). It is important to know the number, size, shape, spatial arrangement, and vertical, lateral, and longitudinal connectivity of non-floodplain wetlands to best quantify how these wetland properties affect watershed state and resilience, and how these effects vary by watershed size, soil and land-use characteristics, near-surface geology, and climatic forces (for example, Mengistu and others 2020). This information can be most efficiently and accurately obtained from interoperable river, stream, and wetland maps built on a common geospatial framework (for example, Johnston and others 2017).
3. *Temporal Fluxes and Interactions* Vulnerable waters are dynamic systems that expand and contract along longitudinal, lateral, and vertical dimensions over time (Stanley and others 1997; Covino 2017; Vanderhoof and others 2018). Research is needed to further characterize the magnitude, frequency, and duration of interactions between vulnerable waters and their upgradient contributing areas (for example, McLaughlin and others 2014; Ward and others 2018), and between vulnerable waters and their down-gradient receiving systems (for example, Ali and English 2019), considering both lateral and vertical interactions and fluxes (for example, Covino 2017).
4. *Thresholds and Drivers of Change* Determining thresholds to alternative watershed states is essential to managing watershed resilience. Knowledge of thresholds provides answers to the often rhetorical question of “how far is too far?” when balancing socioeconomic development targets, with ecological protection and restoration management targets, all the while ensuring maintenance of sustainable ecosystem services. Furthermore, knowledge of thresholds is a benchmark from which to engage with populations and interest groups inured to information about degrading watershed states; identifying a point or threshold beyond which change will occur may empower action. Having determined thresholds affecting state changes, knowledge is needed to determine the priority drivers of change. These are the “levers and pulleys” that can be engaged by societies to adaptively manage a watershed to prevent state change by increasing watershed resilienceLor to try and evince transitions to a new state (for example, Meadows 2008; Zhang and others 2009). For example, is an intervention needed to immediately avoid a state change, or are the thresholds sufficiently distant such that resilient watershed principles can be judiciously applied to avoid an unwelcome transition? Characterizing disturbance drivers (for example, Pascual and Guichard 2005; Radchuk and others 2019) affecting watershed adaptability and resilience allows for interventions to avoid, attenuate, or plan for the coming transition (Scheffer and others 2012). Using these data, resource managers can prioritize watershed management tactics, such as managing and restoring vulnerable waters, to perhaps dampen the likelihood of a transition.
5. *Technical Advances* Increasing hydrological and biogeochemical “big data” and cloud computing availability, analyses, and syntheses presage an improved understanding of vulnerable-water functions, services, and management for resilient watersheds. However, though twenty-first-century models and computational resources are incredibly fast and complex compared to older models, managing the typological interactions and varying fluxes of tens to hundreds of thousands of aquatic system components can still typically overwhelm even these systems. Current solutions require a coarser resampling and forced diminution of the complexities of the spatial fabric to parameterize functioning models (for example, Evenson and others 2018; Driscoll and others 2020). Hence, increased watershed physical representation and vulnerable-water interactions (for example, incorporating vulnerable-water storages, fluxes, and dynamic connectivity) within models requires technical advances in both geostatistical and hydrological modeling applications to incorporate these big data into models (for example, Rajib and others 2020b).
6. *Scale of Influence* While the literature strongly supports the influence of vulnerable waters in determining watershed state and resilience, as we noted above there are limits to discerning “the signal from the noise” (Levin 1992). For instance, watersheds that are naturally or anthropogenically deficient in functioning vulnerable waters will have limited vulnerable-water effects. Where vulnerable waters are more numerous, their cumulative effects may wane with increasing watershed area and the concomitant volumetric mixing and dilution (Sanford and others 2007). And their effects may be obviated with increasing hydrological modification throughout the watershed, such as occurs with the construction of dams, tiling, and artificial drainages (Jones and others 2018; Rajib and others 2020a). Research is thus needed to discern the context and measurable spatial and temporal scale and granularity at which the influence of vulnerable waters is relevant to specific management needs.

## Conclusion

Watersheds are geomorphic structures that receive climatic inputs, delivering a portion thereof to a downstream pour point through both surface water and groundwater flow paths. In the process, materials and energy are transformed and ultimately delivered to down-gradient waters, where ecosystems are supported. Watersheds provide for our most fundamental human needs, including drinking water, clean air, and food resources. Society relies heavily on watersheds’ networked aquatic resources for flood control, navigation, recreation, and aquatic habitat, in addition to the role they play in storing, transforming, or diluting dangerous materials and pollutants.

Yet anthropogenic activities are destroying and degrading native habitats and creating homogeneous landscapes comprised of non-native, mono-cultural vegetation (for example, Foley and others 2005; Levia and others 2020). Part and parcel to this, humans are changing, short-circuiting, and removing hydrogeochemical interactions and altering connectivity (that is, of energy, water, materials, and organisms) within watersheds while concurrently loading aquatic systems with pollutants (Van Cappellen and Maavara 2016; Gramlich and others 2018; Ramankutty and others 2018). An outcome of these watershed-scale modifications includes direct effects on water quantity (for example, Hirsch and Archfield 2015; Mallakpour and Villarini 2015; Peng and others 2019) as well as decreased water quality (for example, Woodward and others 2012).

Watershed resilience provides long-term functional stability in the face of both natural and anthropogenic disturbances. Watershed resilience allows the system to adapt and persist in the face of disturbance without switching to an alternative state that might not maintain crucial ecosystem functions and associated services valued by societies. In the past, watershed resilience appears to have been sufficient to ensure continuity and adaptation of natural systems after all but the most extreme disturbances (for example, large magnitude events with low recurrence intervals, such as volcanic eruptions). In the Anthropocene (Waters and others 2016), however, anthropogenic disturbances have intensified and now operate at such high magnitude and frequency that this has not only led to the loss of individual components, like species, but has caused wholesale changes in the ability of watersheds to regulate themselves, making them more precarious and less resilient to disturbance (for example, Hirota and others 2011; Peterson and others 2021).

Development in watersheds, such as urbanization, increased agricultural intensification, and industrialization, has obvious benefits to society. However, such changes almost always occur piece meal, without integrated planning, and without consideration of the larger, cumulative costs. These costs include not only loss of watershed functions, but also loss of watershed resilience—which leads to instability of watershed functions and inability of a watershed to recover from disturbances.

As we have described here, the individual and cumulative loss of the often unseen and therefore disregarded vulnerable waters reduces watershed resilience. Vulnerable waters substantively affect hydrological and biogeochemical concentrations, storage, and flux variance within and emanating from watersheds. Their loss or degradation, and the loss of the networked interactions between the full extent of the stream network and the landscape draining to it, affects both the individual vulnerable water and cumulative functioning of the watershed. These losses further alter the variance-dampening characteristics and the interactions between watershed components that maintain the resilience of the characteristic system state.

We present four principles for maintaining watershed resilience to hydrological and biogeochemical disturbances vis-à-vis vulnerable waters: (1) Comprehensively map the extent, spatial arrangement, dynamic networked connectivity, and function of vulnerable waters; (2) determine state-changing hydrological and biogeochemical thresholds; (3) identify drivers of change and prioritize management activities; and (4) adaptively manage watersheds. Data availability (that is, measured spatial and temporal connectivity, groundwater and surface water interactions, pollutant sensor data, and so on) features prominently in the identified scientific needs for further quantifying and communicating the importance of vulnerable waters in sustaining and maintaining adaptable and resilient watersheds. Data will dri ve—and adherence to the four principles noted above will guide—the future incorporation of vulnerable waters into scale-appropriate watershed management decisions and will help minimize the loss of vulnerable waters and their cumulative functions that impart watershed resilience.

## Supplementary Material

Supplement1

## Highlights

- Headwaters and non-floodplain wetlands are vulnerable waters
- Vulnerable waters affect hydrologic/biogeochemical processes
- Vulnerable waters adaptively maintain watershed resilience
- Modifications to vulnerable waters induce regime shifts
