# Supplementary material for: Vulnerable Waters are Essential to Watershed Resilience
Source: Ecosystems. Author manuscript; Available in PMC 2023 Aug 2. (PMC10394682; doi:10.1007/s10021-021-00737-2)
Supplement: Supplement1 [file NIHMS1918854-supplement-Supplement1.docx]

**Supplemental Material**

We downloaded the National Wetlands Inventory (NWI) dataset by eight-digit Hydrologic Unit Code (HUC-8) watershed from the US Fish and Wildlife Service Wetlands Mapper (https://www.fws.gov/wetlands) in February and April 2021. NWI data from 2,119 HUC-8 watersheds across the contiguous USA (CONUS) were acquired. The NWI dataset classified wetlands into different systems, including estuarine and marine, riverine, lacustrine, and palustrine (Cowardin and others 1979). Following Lane and D’Amico (2016), we excluded riverine, estuarine, and marine wetlands from analyses as non-floodplain wetlands (NFW). We referred to lacustrine and palustrine wetlands as non-riverine wetlands. Nested lacustrine and palustrine polygons were dissolved and treated as single wetland complexes, which were further used to intersect the NFW centroids at the CONUS scale (Lane and D’Amico, 2016). We then aggregated the area of NFW and non-riverine freshwater wetlands by HUC-8 watershed, respectively. The percentage NFW by HUC-8 watershed was calculated by dividing the total area of NFW (numerator) by the total area of non-riverine freshwater wetlands (denominator). The aggregated areas of NFW and non-riverine freshwater wetlands in CONUS are 116,038 km^2^ and 513,898 km^2^, respectively. The average areal percentage of NFW in CONUS is 22.58% with a range from 0 - 98.83% (standard deviation = 18.18%).

REFERENCES

Cowardin LM, Carter V, Golet FC, LaRoe ET. 1979. Classification of Wetlands and Deepwater Habitats of the United States. Washington DC: US Department of the Interior, Fish and Wildlife Service.

Lane CR, D’Amico E. 2016. Identification of putative geographically isolated wetlands of the conterminous United States. JAWRA Journal of the American Water Resources Association 52:705–722.
